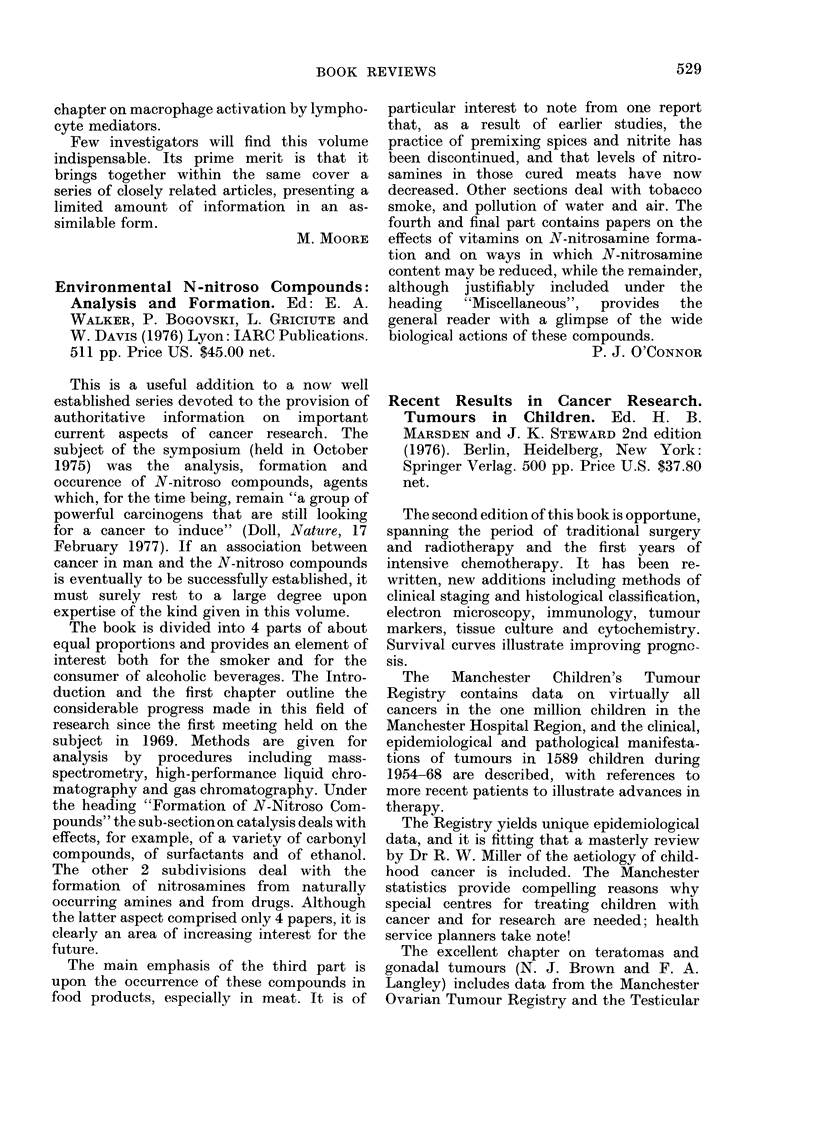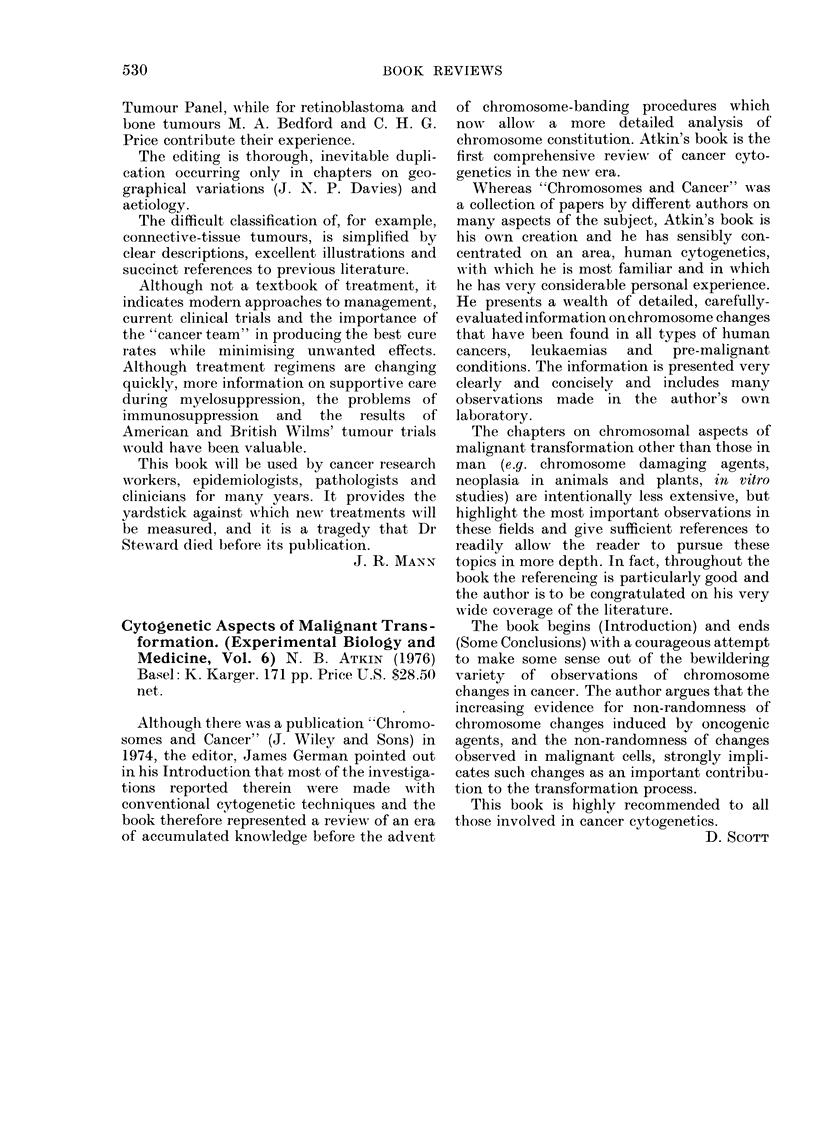# Recent Results in Cancer Research. Tumours in Children

**Published:** 1977-10

**Authors:** J. R. Mann


**Recent Results in Cancer Research. Tumours in Children.** Ed. H. B. MARSDEN and J. K. STEWARD 2nd edition (1976). Berlin, Heidelberg, New York: Springer Verlag. 500 pp. Price U.S. $37.80 net.

The second edition of this book is opportune, spanning the period of traditional surgery and radiotherapy and the first years of intensive chemotherapy. It has been rewritten, new additions including methods of clinical staging and histological classification, electron microscopy, immunology, tumour markers, tissue culture and cytochemistry. Survival curves illustrate improving prognosis.

The Manchester Children's Tumour Registry contains data on virtually all cancers in the one million children in the Manchester Hospital Region, and the clinical, epidemiological and pathological manifestations of tumours in 1589 children during 1954–68 are described, with references to more recent patients to illustrate advances in therapy.

The Registry yields unique epidemiological data, and it is fitting that a masterly review by Dr R. W. Miller of the aetiology of childhood cancer is included. The Manchester statistics provide compelling reasons why special centres for treating children with cancer and for research are needed; health service planners take note!

The excellent chapter on teratomas and gonadal tumours (N. J. Brown and F. A. Langley) includes data from the Manchester Ovarian Tumour Registry and the Testicular

Tumour Panel, while for retinoblastoma and bone tumours M. A. Bedford and C. H. G. Price contribute their experience.

The editing is thorough, inevitable duplication occurring only in chapters on geographical variations (J. N. P. Davies) and aetiology.

The difficult classification of, for example, connective-tissue tumours, is simplified by clear descriptions, excellent illustrations and succinct references to previous literature.

Although not a textbook of treatment, it indicates modern approaches to management, current clinical trials and the importance of the "cancer team" in producing the best cure rates while minimising unwanted effects. Although treatment regimens are changing quickly, more information on supportive care during myelosuppression, the problems of immunosuppression and the results of American and British Wilms' tumour trials would have been valuable.

This book will be used by cancer research workers, epidemiologists, pathologists and clinicians for many years. It provides the yardstick against which new treatments will be measured, and it is a tragedy that Dr Steward died before its publication.

J. R. Mann